# Genome-wide discovery of selection signatures in four Anatolian sheep breeds revealed by ddRADseq

**DOI:** 10.1038/s41598-024-71617-7

**Published:** 2024-09-03

**Authors:** Bahar Argun Karsli, Eymen Demir, Umit Bilginer, Huriye Dogru, Taki Karsli, Sarp Kaya

**Affiliations:** 1 Department of Agricultural Biotechnology, Faculty of Agriculture, Eskişehir Osmangazi University, Eskişehir, 26160 Türkiye; 2 Department of Animal Science, Faculty of Agriculture, Akdeniz University, Antalya, 07070 Türkiye; 3 Department of Animal Science, Michigan State University, East Lansing, MI 48824 USA; 4 Department of Medical Services and Techniques, Vocational School of Burdur Health Services, Burdur Mehmet Akif Ersoy University, Burdur, 15100 Türkiye; 5 Institution Department of Animal Science, Faculty of Agriculture, Eskisehir Osmangazi University, Eskisehir, 26160 Türkiye

**Keywords:** ddRADseq, NGS, Selection signals, Selection sweeps, Turkish sheep, Next-generation sequencing, Animal breeding, Genetic association study

## Abstract

High-density genomic data analyzed by accurate statistical methods are of potential to enlighten past breeding practices such as selection by unraveling fixed regions. In this study, four native Turkish sheep breeds (80 samples) were genotyped via 296.097 single nucleotide polymorphisms (SNPs) detected by double-digest restriction site-associated DNA (ddRADseq) library preparation combined with the Illumina HiSeq X Ten instrument in order to identify genes under selection pressure. A total of 32, 136, 133, and 119 protein-coding genes were detected under selection pressure by runs of homozygosity (ROH), integrated haplotype score (iHS), the ratio of extended haplotype homozygosity (Rsb), and fixation index (F_ST_) approaches, respectively. Of these, a total of 129 genes were identified by at least two statistical models which overlapped with a total of 52 quantitative trait loci (QTL)-associated SNPs, known to be related to fiber diameter, milk content, body weight, carcass traits, some blood parameters, and entropion. A total of six genes under selection pressure were validated by three statistical approaches five of which are of potential to be integrated into animal breeding since they were associated with wool fiber diameter (*ZNF208B*), behaviors related to neurocognitive development (*CBX1* and *NFE2L1*), adaptation to high-altitude (*SDK1*), and anxiety causing internal stress (*GSG1L*). The sixth gene (*COPZ1*) turned out to play an important role in coping with different types of cancer in mammals. In particular, ROH analysis uncovered significant findings that the Güney Karaman (GKR) had experienced different selection practices than the Akkaraman (AKR) breed. Moreover, some genes specifically under selection in the GKR breed turned out to be associated with olfaction (*OR6K6, OR6N1, OR6N2,* and *OR4C16*), survival during the gestation period (*PRR15L*), and heat stress (*CDK5RAP9*). The results of this study imply that GKR may become genetically different from the AKR breed at the genome level due to most probably experiencing different adaptation processes occurring in raised climatic conditions. These differences should be conserved to face future challenges, while other native Turkish sheep breeds could be monitored via genome-wide high-density SNP data to obtain deeper knowledge about the effects of natural selection.

## Introduction

The sheep (*Ovis aries*) were domesticated for wool and meat production around 11,000 years ago. According to archaeological excavations and bone remnants, the process of sheep domestication unfolded in a region stretching from northern Zagros to southeastern Anatolia, commonly known as the Fertile Crescent^1^. Since domestication to the present day, sheep have been utilized not only for acquiring animal products like meat and milk but also for crafting various items (clothing, blankets, bows, etc.) to address environmental challenges^2^. Domestication of sheep, selected by herders and breeders based on economic and physical traits, has significantly contributed to the emergence and development of modern sheep breeds. Today, there are more than 1.400 distinct sheep breeds around the globe^3,4^. Being a part of the sheep domestication center, Türkiye significantly contributes to the global sheep biodiversity with approximately 42 million sheep and nearly 30 different local breeds or types^5,6^.

In the realm of sheep breeds nurtured in Türkiye, the AKR takes the lead with an overwhelming preference, accounting for about 40%. The prevalent preference for the AKR breed, possessing fat-tailed phenotype, in the central region of Türkiye, arises from its exceptional acclimatization, survival in challenging environmental conditions, and success in utilizing most insufficient pasture conditions^7,8^. Being raised in the eastern and northeastern provinces of Türkiye and constituting approximately 15% of the sheep population, Morkaraman (MKR) is recognized for its ability to adapt to the harsh conditions specific to nomadic lifestyle and stand out as an indispensable element of transhumance with their capacity to thrive in large pastures, mountainous and rural areas^9,10^. Karakaş (KRK) is reared in the Lake Van basin with high altitude conditions by family farms at a small-scale production for not only meat and milk but also for fleece yield^11^. GKR sheep, characterized by its fat tail phenotype and distinctive coat colour, has been traditionally bred in the Taurus Mountains of the Mediterranean Region (especially in the provinces of Adana, Mersin, and Antalya) for many years, but due to non-systematic crossbreeding practices, the GKR is currently on the verge of rapidly endangered breeds. Indeed, a decreased trend in purity and population size of GKR breed has forced the Turkish Ministry of Agriculture and Forestry to take precautions to initiate a conservation program at the Bahri Dagdas International Agricultural Research Institute back in 2001^12^. Despite conservation efforts, it has been stated in various studies that the GKR sheep remains under threat and has not yet reached a sufficient population size^8,13–15^.

For many years, both natural and artificial selection pressure has been imposed on sheep for adaptation to environmental challenges as well as economic and morphological characteristics in line with the preferences of human beings. Genomic regions subjected to intensive selection processes for the long term exhibit increased linkage disequilibrium (LD), homozygosity, and reduced genetic diversity^16,17^. According to the theory of population genetics, functional genes exposed to selection pressure reveal characteristic patterns called "selection signatures"^18^. Mutations that provide an advantage regarding many aspects from survival to economically important traits can be determined using selection signatures. Indeed, by utilizing genome-wide genetic data, previous studies on various sheep have reported signals of selection in numerous regions related to reproduction^19^, body weight^20^, milk yield^21^, wool traits^22^, immunity and disease^23^, thermo-tolerance^24^, and adaptation to high altitude^25^.

Recent developments in DNA sequencing technology have made it possible to identify and genotype SNPs across numerous individuals simultaneously^26^. SNPs-based genotyping is commonly used to assess selection signatures and genetic diversity since they are genetically stable, abundant, and randomly distributed throughout the genome. Several statistical approaches are available to unfold genomic regions under selection pressure by within-population and between-population statistical models. Furthermore, Liu et al.^27^ have underscored that the combination of multiple statistical approaches is beneficial in unveiling footprints and enhancing the identification power and spatial resolution of selection signatures. The iHS approach, which is based on LD and is an extension of the extended haplotype homozygosity (EHH) method, has been developed to avoid the influence of heterogeneous recombination rates across the genome^28^. iHS is commonly used to detect information from a single population and species-specific genes under positive selection^29,30^. The ratio of EHHs between populations, based on LD and utilized as a complement to iHS for pairwise population comparisons (Rsb), is an approach employed to pinpoint recent selective sweeps resulting in the near or complete fixation of an allele within a population^31,32^. Autozygous stretches, also referred to as ROH, allow for the detection of potential selection signatures for the breed^33^. Additionally, the frequency and size of ROH vary according to population diversity and selection pressure^27^. The F_ST_ approach, based on differences in allele frequencies, is a widely used statistical method capable of detecting candidate genes between populations^34^.

Thanks to protocols such as restriction site–associated DNA (RAD) sequencing^35^, genotyping-by-sequencing (GBS)^36^, and ddRADseq^37^, which combine the strength of next-generation sequencing (NGS) and are known as restriction site-associated DNA sequencing (RADseq) techniques, subsets of the genome sequence are obtained^38^. These methods, initially developed for different species, have also been implemented for farm animals such as cattle^39^, sheep^10^, goats^40^, pigs^41^, and buffalo^42^. Compared to the RADseq method, the ddRADSeq, a cost-effective technique of reduced representation of the genome, benefits from two restriction enzymes to reduce genome complexity for SNP discovery and genotyping.

To our best knowledge, no studies have been published in the literature to investigate signatures of selection in native Turkish sheep breeds, whereas Türkiye is a part of the domestication center of *Ovis aries* and has a significant potential to enlighten the evolution process of sheep genome. Moreover, ddRADseq data have never been used to investigate selection signals in sheep worldwide. Therefore, by adopting the ddRADseq method combined with Illumina HiSeq X Ten platform, this is the first comprehensive study to detect genomic regions under selection pressure by both within (ROH and iHS) and between (F_ST_ and Rsb) populations approaches which could explain phenotypic differences among four Anatolian sheep populations.

## Results

In this study, a total of 4.730.879 SNPs and 286.099 InDels were detected across 80 animals at the variant calling process. During the filtering process, however, a total of 296.097 SNPs with high call rates across 74 individuals (2 and 4 samples from AKR and KRK were discarded due to low genotyping rate) were retained to detect selection signatures.

**Fig. 1:**
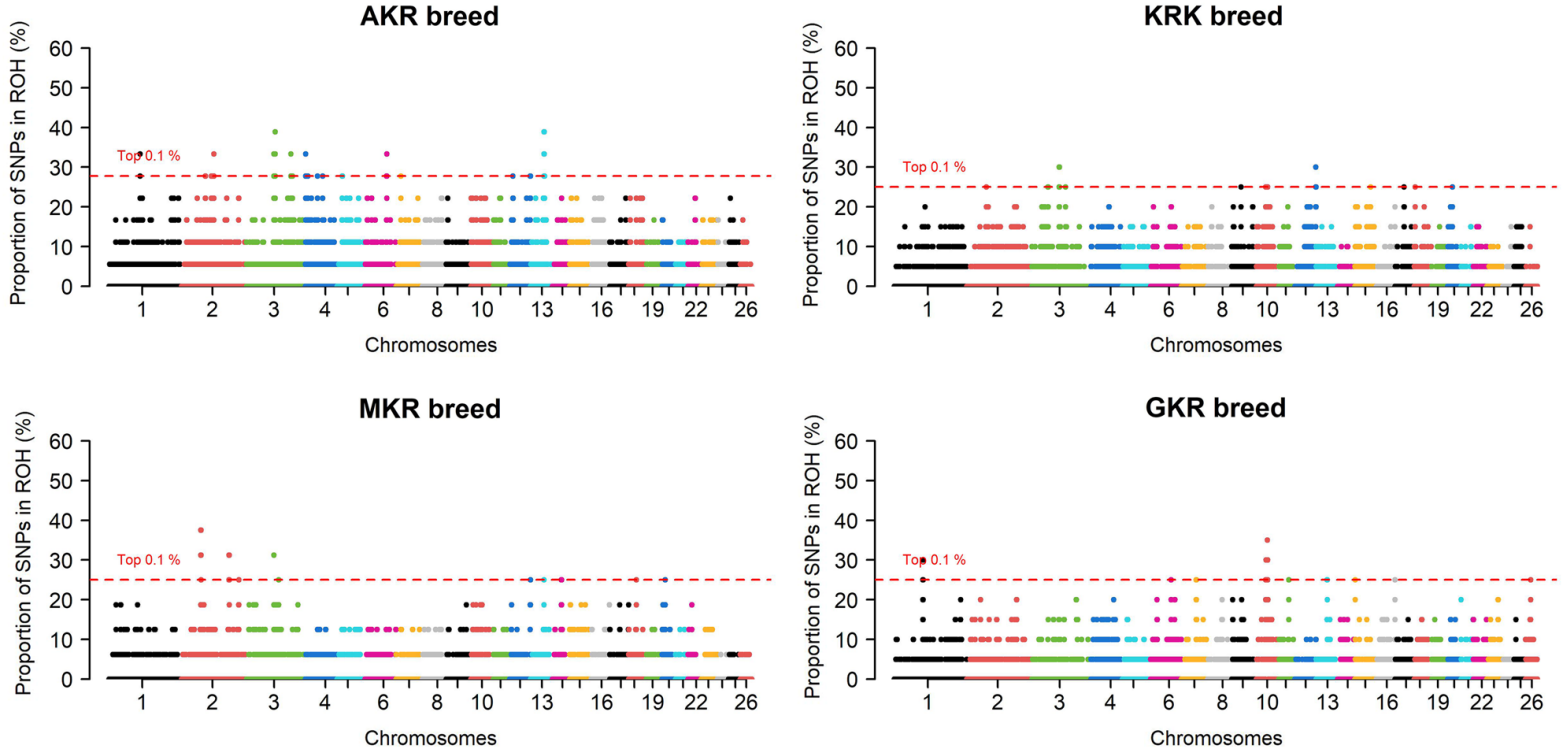
Manhattan plot of ROH based selection signals among four sheep populations.

**Fig. 2:**
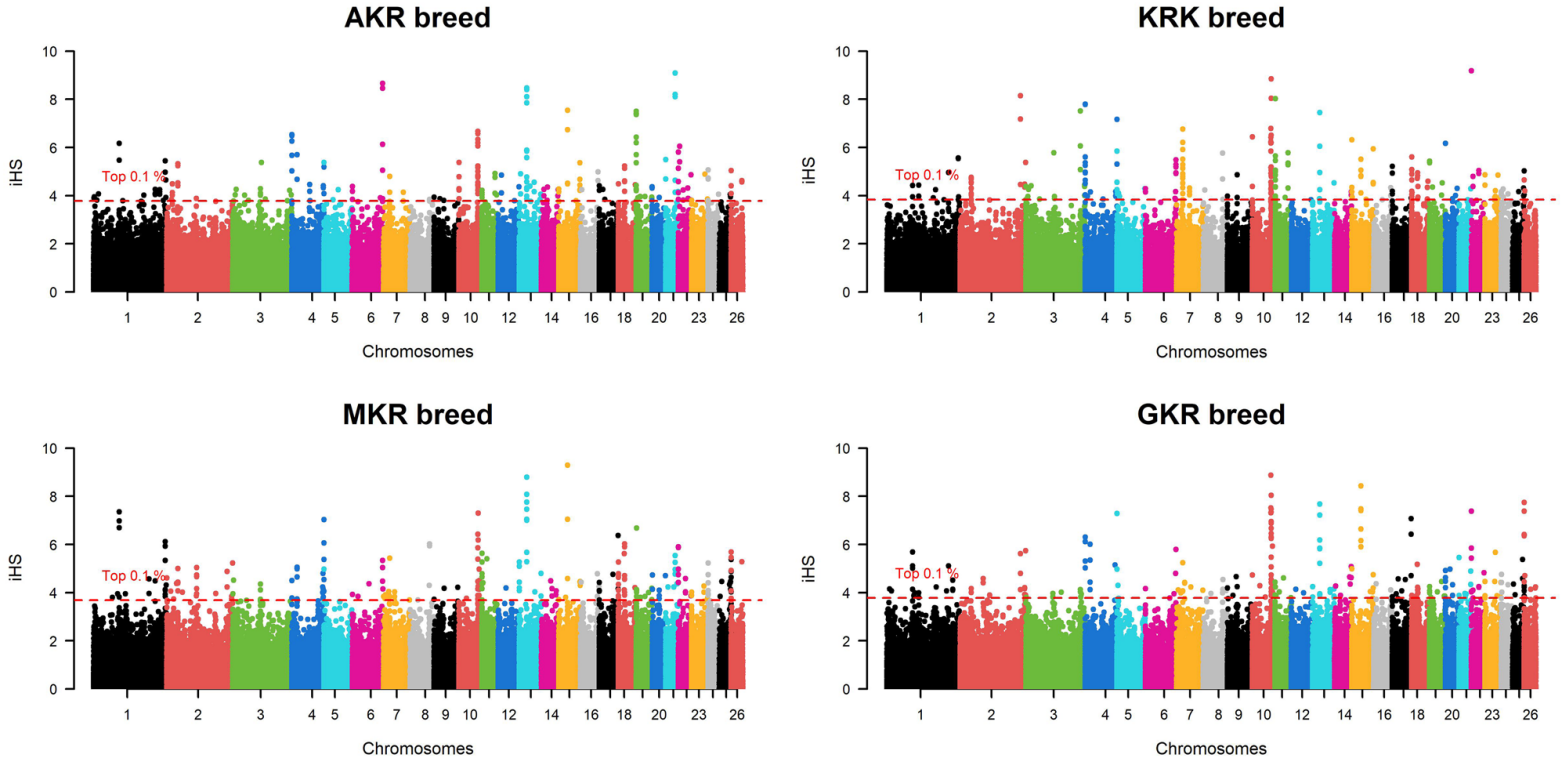
Manhattan plot of iHS based selection signals among four sheep populations.

**Fig. 3:**
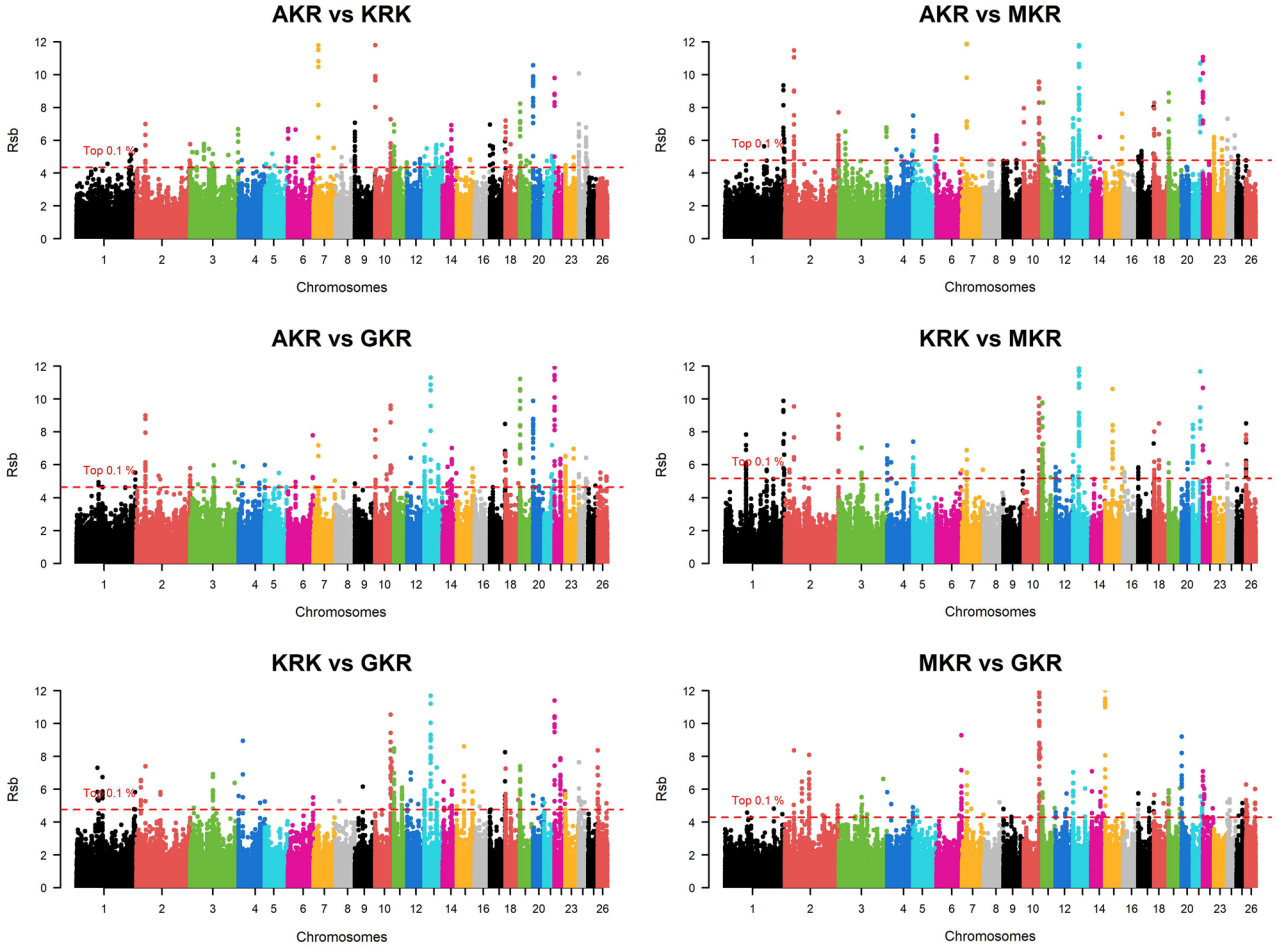
Manhattan plot of Rsb-based comparative selection signals among four sheep populations.

**Fig. 4:**
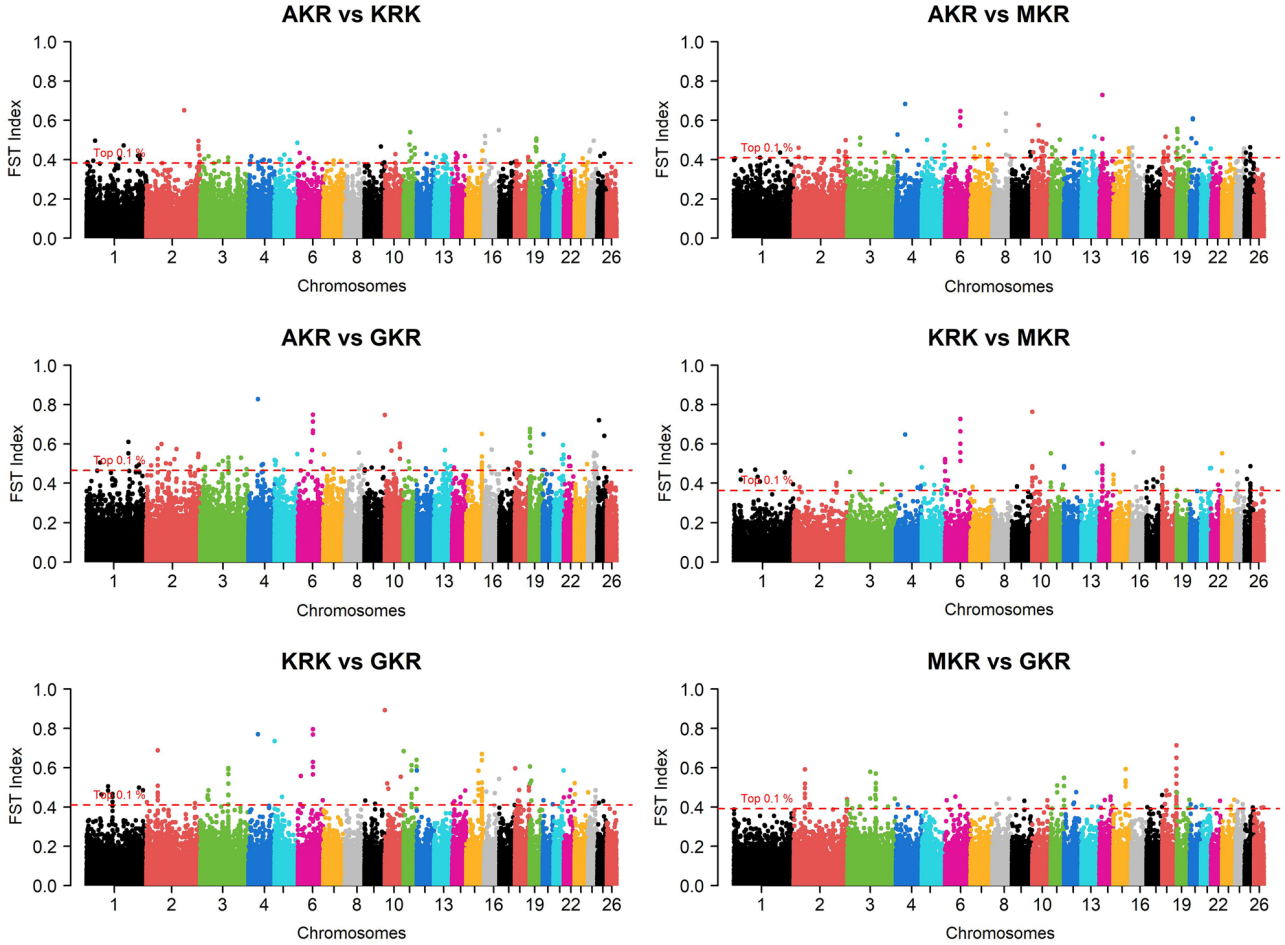
Manhattan plot of FST-based comparative selection signals among four sheep populations.

SNPs were visualized in a Manhattan plot based on their calculated ROH frequency (Fig. 1), iHS (Fig. 2), Rsb (Fig. 3), and F_ST_ (Fig. 4) values in which only the top 0.1% SNPs were chosen according to empirical distribution.

**Fig. 5:**
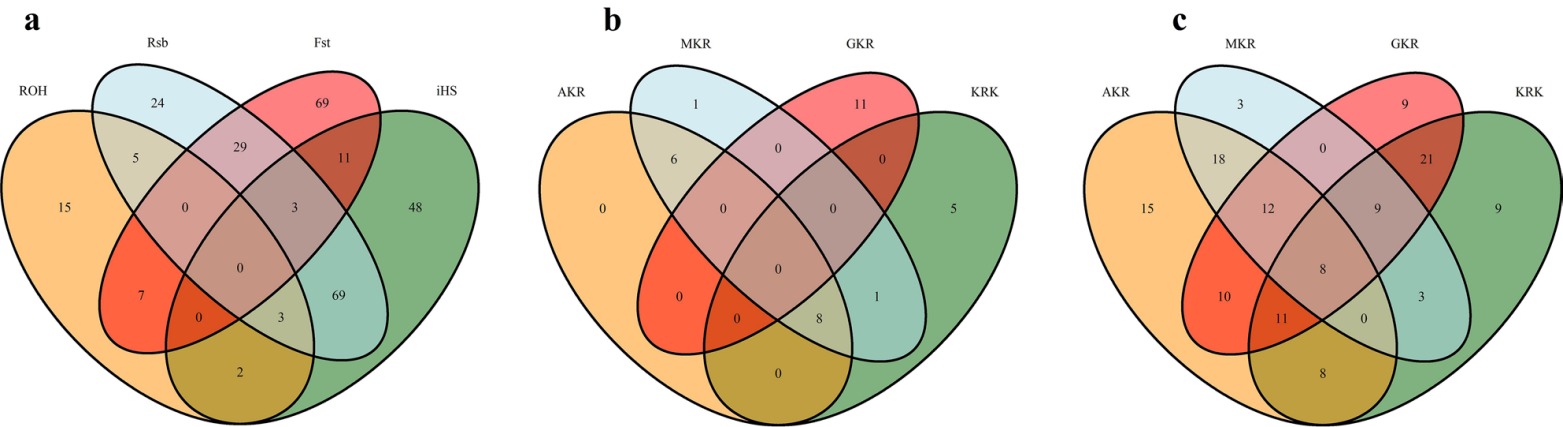
Venn diagram for number of genes detected by (**a**) four statistical approaches per all population and breed-level for (**b**) ROH and (**c**) iHS statistics.

When approaches were analyzed individually, selection pressure was observed in 285 different protein-coding genes of which 32, 136, 133, and 119 genes were detected by ROH, iHS, Rsb, and F_ST_, respectively (Fig. 5a). Detailed information such as chromosome, start position, end position, as well as effects on the phenotype of these selection signals were summarized (Supplementary Data 1). Some common genes were detected within population statistics such as ROH (Fig. 5b) and iHS (Fig. 5c). In ROH approach, for example, a total of eight protein-coding genes (*ACOXL*, *BCL2L11*, *PPP1R12B*, *UBE2T*, *LGR6*, *PTPN7*, *ARL8A*, and *GPR37L1*) turned out to be under selection pressure in AKR, KRK, and MKR breeds (Supplementary File 1), whereas these genes showed no pattern of selection in GKR breed. Similarly, 11 protein-coding genes (*OR6K6*, *OR6N1*, *OR6N2*, *CBX1*, *NFE2L1*, *COPZ2*, *CDK5RAP3*, *PRR15L*, *PNPO*, *SP2*, and *OR4C16*) were under selection pressure in the GKR breed, while these genes were not detected by ROH statistics in AKR, KRK, and MKR breeds. No genes specific to the AKR breed were detected by ROH, while *CDH3* was under selection pressure only in MKR as well as *ZDHHC20*, *SAP18*, *LATS2*, *XPO4*, and *SUPT3H* only in the KRK breed (Supplementary File 1). In the iHS approach, on the other hand, a total of eight genes (*R3HCC1*, *CHMP7*, *RHOBTB2*, *ACOX3*, *TRMT44*, *FAM110B*, *GRAMD1B*, and *OR5A1*) were under selection pressure in all breeds (Supplementary File 1). As observed in ROH statistics, some genes (*PDE4DIP*, *SCYGR4*, *PLA2G5*, *PLA2G2E*, *OTUD3*, *DOK5*, *ZNF280B*, *WNT5A*, *COG2*, *BEND7*, *PRPF18*, *FRMD4A*, *OR6Q1*, *OR9I1*, *OR10Q1*, *ACAD9*, *CFAP92*, *GP9*, *RAB43*, *ISY1*, *UBE2E2*, *LOXL2*, *TSEN34*, *MBOAT7*, *TMC4*, *LENG1*, *CNOT3*, *PRPF31*, *TFPT*, *OSCAR*, *TARM1*, and *MBL2*) were common among AKR, KRK, and MKR breeds, while a total of eight genes (*FOXN3*, *ABCA10*, *ABCA6*, *ABCA9*, *WWOX*, *OR8K1*, *RYR2*, and *ZP4*) under selection were specific to only in GKR breed (Supplementary File 1). *R3HCC1*, *CHMP7*, and *RHOBTB2* genes turned out to be under selection pressure in all breeds via the Rsb approach. No genes under selection pressure across all populations were observed via the *F*_*ST*_ approach. Besides, numerous genes were found to be differently fixed between AKR and other breeds according to iHS (*ACOX3*, *TRMT44*, *CPZ*, *FREM3*, *SMARCA5*, *GAB1*, *SMCHD1*, *EMILIN2*, and *LPIN2*), while F_ST_ statistics generally monitored the genes differently under selection between KRK and other breeds.

However, in order to eliminate false positive results, we considered selection signatures in the genes represented by at least two statistical methods. In this regard, 129 of 285 genes were detected to be under selection pressure by at least two statistical models. A large part of these genes (72) were common between Rsb and iHS approaches, while only five common genes (*CBX1*, *NFE2L1*, *COPZ2*, *OR4C16*, and *SUPT3H*) were observed between ROH and iHS statistics. No genes were detected by four statistical approaches, while selection pressure was observed in 6 genes (*CBX1*, *NFE2L1*, *COPZ2*, *ZNF280B*, *SDK1*, and *GSG1L*) via three statistical models. These selection signatures overlapped with 52 QTL-associated SNPs some of which were directly related to fiber traits (*PIK3R4*, *COL6A6*, and *COL6A5*), milk content (*LOXL2*, *R3HCC1*, *CHMP7*, *RHOBTB2*, *PEBP4*, *ARHGEF10L*, and *FAM110B*), body weight (*SLCA6A2*, *MT3*, and *MT4*), carcass traits (*TSHZ2*, *CCDC12*, *PTH1R*, *MYL3*, *PRSS42P*, *MBL2*, *KATNIP*, and *GSG1L*), some blood parameters (*OR6K6*, *OR6N1*, *OR6N2*, *BEND7*, *PRPF18*, *FRMD4A*, and *IFITM10*), and an ocular disease known as entropion (*OR8K1* and *OR5A1*).

## Discussion

In this study, a total of 32, 136, 133, and 119 protein-coding genes were detected to be under selection pressure via ROH, iHS, Rsb, and F_ST_ statistics, respectively, in four Anatolian sheep populations. The number of genes detected by iHS, Rsb, and F_ST_ were significantly higher than the genes detected by ROH. This could be explained by the differences between assumptions under these approaches in which ROH is of more strict criteria resulting in screening a lower number of intervals across the genome. Similar findings were also reported by previous studies. For example, three Indian native sheep breeds (Changthangi, Deccani, and Garole) were genotyped by the Ovine SNP50 BeadChip to investigate genetic diversity and selection signatures in which a higher number of genes were detected by iHS (140) compared to ROH (48) statistics^54^. Another study conducted by Moravčíková et al.^17^ showed a higher number of genes detected under selection by F_ST_ (1604) compared to the ROH (from 52 to 866) approach in six beef cattle breeds (Aberdeen Angus, Hereford, Limousin, Charolaise, Piedmontese, and Romagnola) genotyped with BovineSNP50v1 BeadChip array.

Selection signature analyses are efficient in revealing genomic regions exposed to natural and artificial selection, while they do not require phenotypic data and could be easily investigated via SNP data obtained from different platforms such as array and NGS technologies. In Türkiye, however, identifying selection signatures is a new field of study due to the fact that native livestock species have been mainly genotyped by microsatellite markers and single genes. Indeed, only one published study was detected by checking literature in which Demir et al.^39^ monitored signatures of selection in six native Anatolian and two cosmopolitan cattle breeds via 211.119 bi-allelic SNPs by adopting three statistical approaches. Unfortunately, no published studies on revealing genomic regions under selection pressure in native Turkish sheep breeds were available in the literature. Therefore, the current study is of significant importance in obtaining deeper knowledge of past selection practices in native Turkish sheep breeds. Indeed, in this study, selection pressure on 129 protein-coding genes was detected at least by two statistical models. These genes turned out to include a total of 52 QTL-associated SNPs which were related to several traits such as fiber diameter, milk content, body weight, carcass traits, some blood parameters, and entropion. Most of the genes (123 genes) were detected by two statistical approaches, whereas six genes (*CBX1*, *NFE2L1*, *COPZ2*, *ZNF280B*, *SDK1*, and *GSG1L*) were identified by three statistical models indicating that they are of potential to enlighten selection process in Anatolian sheep breeds. Therefore, previous studies were checked to obtain more knowledge about the effects of these genes on phenotype. As highlighted by Dementieva et al.^55^, the *CBX1* gene is associated with neurocognitive development in humans. Similarly, a recent study confirmed that mutation in the *CBX1* gene could cause a novel syndromic neurodevelopmental disorder in mice^56^. The expression profile of the *NFE2L1* gene is related to response to oxidative stress in living cells. A study showed that since *NFE2L1*-*PBX1* is a novel neuroprotective pathway in humans, it could be utilized to formulate new drugs to treat Parkinson's Disease^57^. Another study conducted by Trukhachev et al.^58^, however, confirmed that polymorphisms in the *NFE2L1* gene were associated with meat production indicators in sheep. Regarding the *NFE2L1* gene, the results of the current study are relevant to a documentary released by the General Directorate of Agricultural Research And Policies (GDARP). Indeed, selection pressure on the *NFE2L1* gene was detected in GKR and KRK breeds by ROH and iHS approaches, respectively. This finding proves that although GKR and KRK have been derived from the AKR breed, they may have experienced different breeding practices in the past. Similarly, it is documented that GKR and KRK show significant differences from the AKR breed in terms of body weight^59^. This finding implies that the *NFE2L1* could be subjected to selection programs as a candidate gene to improve carcass traits in Anatolian sheep populations. The *COPZ2* gene is known to be associated with different types of cancer. For example, Shtutman et al.^60^ articulated that the *COPZ1* gene is of potential to kill cancer cells when the *COPZ2* gene is down-regulated. Therefore, it is pointed out that the expression level of the *COPZ2* gene could be decreased via silencing microRNA 152 and *COPZ1*-targeting agents could be deployed to selectively kill different cancer cells^60^. A competitive allele-specific PCR-based study on 1081 Alpine Merino sheep revealed that variations of the *ZNF208B* gene are directly associated with wool fiber diameter^61^. Since fiber diameter is one of the most important characteristics of wool, the *ZNF208B* is of potential to be subjected to selection studies by the farmers. In this study, selection signals were detected for the *ZNF208B* gene in GKR, MKR, and KRK breeds via the iHS. Besides, the Rsb approach confirmed that this gene has been differently fixed between AKR and the other three breeds. Unfortunately, no studies were detected in the literature to compare wool characteristics among four studied sheep breeds. Therefore, the results of the current study regarding the *ZNF208B* gene require confirmation by further studies to create a basis for selection programs to improve wool traits. As highlighted by Wiener et al.^62^, the *SDK1* gene plays an important role in adaptation to the environment in which variations near this gene were reported to be associated with oxygen saturation in humans living in regions with high-altitude in Ethiopia and adaptation against high altitude/low temperature in Ethiopian chicken^63,64^. No studies directly focusing on *GSG1L* gene sheep were detected in the literature, while a significant correlation between the expression level of *GSG1L* and composite anxiety level was reported in mice^65^.

Although no detailed information about the genetic origin of native Turkish sheep is available, GKR was considered as a variety of AKR breed for a long time. On the contrary, this information is questioned among scientists nowadays because these populations possess different phenotypic traits and reared in different regions with diverse adaptation processes for centuries. A recent study based on 21 microsatellite markers revealed for the first time that GKR has become genetically different from the AKR breed according to population structure and phylogenetic analyses^8^. Moreover, the authors highlighted that this difference should be confirmed by further comprehensive studies. As recommended by Karsli et al.^8^ significant results were obtained via the ROH approach in this study using high-density SNP data. In this study, 6 and 8 genes under selection pressure were common between AKR-MKR and AKR-KRK, whereas no common genes were identified between AKR and GKR populations. A total of 11 genes were unique to GKR 6 of which (*OR6K6*, *OR6N1*, *OR6N2*, *OR4C16*, *PRR15L*, and *CDK5RAP9*) are of potential to enlighten the past breeding history of the GKR breed. Of these unique genes, *OR6K6*, *OR6N1*, *OR6N2*, and *OR4C16* were associated with olfactory systems in mammals. Similarly, Demir et al.^39^ revealed that two genes associated with olfaction (*OR4C1F* and *OR4C1E*) were under selection pressure in eight cattle breeds reared in Türkiye. Olfactory is one of the major systems for animals to survive since as documented by Wackermannová et al.^66^, it allows for detection and discrimination of home range, mates, food resources, predators, and prey around. On the other hand, recent studies confirmed that *PRR15L* also plays an important role in mammals by enhancing trophoblast viability and survival during the gestation period, especially in early implantation and placentation^67^, while *CDK5RAP9* was reported to be associated with heat stress in sheep^68^.

This study demonstrates that even when phenotypic records are not available, high-density SNP data are of potential to reveal significant clues about past breeding practices causing breed differentiation regarding related genomic regions. Although MKR, KRK, and GKR are believed to be derived from AKR breed, as articulated by Karsli^10^, these varieties show differences compared to AKR breed in terms of morphological and economically important traits due to being raised in different climatic zones with diverse altitudes and temperatures for centuries. In this study, the results of ROH in particular were consistent with geographical distributions and breeding history of the studied sheep populations. This approach uncovered that a total of 14 and 8 protein-coding genes under selection pressure were in common between AKR-MKR and AKR-KRK, respectively, while no common genes between AKR and GKR were identified. Besides, a total of 11, 5, and 1 protein-coding genes under selection turned out to be specific to GKR, KRK, and MKR breeds, respectively. Detection of no common selection signatures between AKR and GKR is not surprising due to the fact that Taurus Mountains are located between their rearing climatic zones making a geographical isolation for GKR breed. Due to rearing regions of GKR, Antalya and Mersin provinces which are notorious for their higher temperature and humidity, it is not surprising to identify selection signals in heat and heat-related protein-coding genes such as *CDK5RAP9*. On the other hand, detection of the highest and lowest number of common fixed genomic regions between AKR-MKR and AKR-KRK is consistent with their breeding history because AKR and MKR have been reared in Central Anatolia for centuries, whereas KRK survives in a limited and isolated geographic region (Van province) thanks to efforts of smallholder farmers. The results of this study were also convenient with another climatic condition known as altitude which significantly varies from one region to another in Anatolia. It is known that GKR and KRK are raised in regions with low and high altitudes, respectively. Indeed, Rsb and F_ST_ approaches revealed that *SDK1* gene, associated with adaptation to high/low altitudes, were differently fixed between AKR-KRK and AKR-GKR breeds.

## Conclusion

While advanced molecular genotyping methods and accurate statistical analyses offer new opportunities to obtain deeper knowledge of past breeding practices such as selection in farm animals, it is still a new research area when it comes to native Turkish livestock species. Hence, selection signatures in four Anatolian sheep breeds were analyzed via high-density genomic data (296.097 SNPs) for the first time. Selection pressure on 129 protein-coding genes overlapped with 52 QTL-associated SNPs. The sheep QTL database confirmed that these SNPs were previously reported to be associated with fiber diameter, milk content, body weight, carcass traits, some blood parameters, and entropion. Besides, a literature-based investigation confirmed that six genes detected by three statistical approaches were related to wool fiber diameter, neurocognitive development, numerous cancers, adaptation to high altitude, and anxiety. One of the most interesting results was that some genes were common between AKR, MKR, and KRK via the ROH approach, while no common genes between AKR and GKR were identified. This finding is quite novel since GKR is considered as a variety of AKR breeds indicating that they should have experienced the same selection process. However, these breeds show differences in terms of several phenotypes as well as being raised in different climatic zones for centuries. This study demonstrated that genome-wide high-density data processed by accurate statistical analyses may reveal differences in terms of past breeding practices in livestock breeds. On the other hand, some genes related to survival traits (*PRR15L*), olfaction (*OR6K6*, *OR6N1*, *OR6N2*, and *OR4C16*), and heat stress (*CDK5RAP9*) were identified in the GKR breed indicating that special importance should be given to GKR breed in conservation studies to maintain animal production against face future challenges such as climate change. Since the results of this study were novel and covered 20 representative samples per breed, we encourage further studies to focus on revealing fixed genomic regions in other native Turkish sheep breeds with higher sample sizes per breed which could be genoytped by more sophisticated sequencing platforms such as NovaSeq to obtain higher resolution SNP data. Moreover, identified selection signatures could be analysed through genome-wide association analysis by further studies utilising phenotypic records.

## Methods

### Sampling strategy, genomic library preparation, and Illumina sequencing

A total of 80 animals belonging to AKR (n = 20), MKR (n = 20), GKR (n = 20), and KRK (n = 20) breeds were sampled from representative herds reared in Konya, Ağrı, Van, and Antalya provinces, respectively. GeneJET Genomic DNA Isolation Kit (Thermo K0721) was performed to isolate DNA from blood samples according to the manufacturer's guidelines. The presence of DNA was validated via agarose gel electrophoresis, while the quantity was identified by Qubit 4™ fluorometer (ThermoFischer Scientific) with a double-stranded DNA HS assay kit (Invitrogen). The standart ddRADseq pipeline recommended by Peterson et al.^37^ was exploited to prepare DNA libraries for the sequencing process. *EcoR*1 and *Msp*I enzymes were utilized to digest total DNA followed by the ligation of the P1 adapter. Further, all DNA fragments were pooled, sheared, and P2 adapters were fused via T4 DNA ligase. DNA libraries were enriched by the PCR method after DNA fragments with 300–500 bp length were selected. DNA libraries prepared according to ddRADseq were subjected to the Illumina Hiseq X Ten platform to obtain short reads.

### Genomic data processing and variant calling

Based on their adapter information, short reads were assigned to individuals via the *process_radtags* command implemented in the Stacks 2 program^43^. Demultiplexed reads were filtered based on adapter sequences and reads with low-quality and ambiguous bases via the fastp program^44^ to obtain clean reads. Bowtie2 algorithm^45^ with default parameters was run to align clean reads to the *Ovis aries* reference genome (ARS-UI_Ramb_v3.0). Genomic data were further subjected to BCFtools^46^ pipeline for variant calling in which only biallelic SNPs with high read depth (20 ≤ D ≤ 500) and quality (Q ≥ 20) were considered. All InDels together with the SNPs located on sex chromosomes and mtDNA were removed from the dataset. The remaining SNPs were filtered in terms of minor allele frequency (MAF > 0.05) and the genotyping rate of 90% across the individuals by the PLINK 1.9 program^47^. Further, individuals with a lower genotyping rate of 90% in terms of the remaining SNPs were filtered to obtain the final dataset.

### Detection of genome-wide selection signatures

It is noteworthy that several statistical approaches are available to detect signatures of selection within and between populations, while as highlighted by Liu et al.^48^, each method comes with some limitations. Therefore, in this study, four complementary statistical approaches (ROH, iHS, F_ST_, and Rsb) were considered to eliminate false positive results caused by these limitations. ROH and iHS were used to investigate the selection of signatures within each breed, while F_ST_ and Rsb approaches were considered to detect differently fixed (or close to fixation) between studied populations.

ROH statistics-based selection signatures were identified within each sheep breed via the detectRUNS package^49^ with a consecutive runs approach. A common criterion was used to define ROH segments in all sheep breeds as follows: (i) the minimum number of consecutive SNPs per each ROH island was adjusted as > 50, (ii) the minimum length of a ROH was set to 0.5 Mb, (iii) the maximum gap between consecutive homozygous SNPs was 1 Mb, (iv) the maximum two SNPs with missing genotypes and v) one heterozygous call were allowed in an ROH.

Before screening each breed in terms of iHS statistics, the SNPs were phased in chromosome-wise by the Beagle 5.4 program^50^ with default parameters. The iHS values per each SNP across the populations were computed via the *rehh* package^51^.

Unlike the ROH and iHS approaches, each breed was compared to other populations in terms of F_ST_ and Rsb statistics. Pairwise F_ST_ values per SNPs were computed by using PLINK 1.9 software^47^, while Rsb values were calculated via the *rehh* package^51^ following chromosome-wise haplotype phasing performed by Beagle 5.4 program^50^ with default parameters. PLINK 1.9^47^, detectRUNS^48^, and the *rehh*^51^ packages were run in the R environment^52^.

### Detection of SNP outliers and gene annotation

The Manhattan plots at genome-wide level for ROH, iHS, F_ST_, and *Rsb* values were visualized by the *qqman* package^53^. Strict criteria were applied to define outlier SNPs in order to reduce false positive outputs. Firstly, the top 0.1% SNPs with the highest ROH, iHS, F_ST_, and Rsb values according to empirical distribution were considered, while the remaining SNPs were neglected. Secondly, genomic windows of non-overlapping 200 kb (100 kb upstream and 100 kb downstream) were screened to limit the genetic interval of outlier SNPs. Finally, as adopted by Liu et al.^48^, potential candidate regions detected by at least two methods were considered to be under selection in order to reduce the false-positive outputs. Protein-coding genes within these candidate regions were identified by BIOMART (https://www.ensembl.org/info/data/biomart) with the option of ARS-UI_Ramb_v2.0 assembly, whereas their effects on phenotype were confirmed based on sheep QTL database (https://www.animalgenome.org/cgi-bin/QTLdb/OA).

## Supplementary Information

Supplementary Table 1.

## Data Availability

The datasets analysed during the current study are available for academic purposes upon signing a Material Transfer Agreement with the corresponding author (baharargunkarsli@gmail.com).
